# The Impact of Exercise Training and Supplemental Oxygen on Peripheral Muscles in Chronic Obstructive Pulmonary Disease: A Randomized Controlled Trial

**DOI:** 10.1249/MSS.0000000000003268

**Published:** 2023-08-02

**Authors:** DANIEL NEUNHÄUSERER, MARTIN HUDELMAIER, DAVID NIEDERSEER, MARCO VECCHIATO, WOLFGANG WIRTH, EVA STEIDLE-KLOC, BERNHARD KAISER, BERND LAMPRECHT, ANDREA ERMOLAO, MICHAEL STUDNICKA, JOSEF NIEBAUER

**Affiliations:** 1 University Institute of Sports Medicine, Prevention and Rehabilitation, Paracelsus Medical University of Salzburg, Salzburg, AUSTRIA; 2 Research Institute for Molecular Sports Medicine and Rehabilitation, Paracelsus Medical University of Salzburg, Salzburg, AUSTRIA; 3 Institute of Anatomy and Cell Biology, Paracelsus Medical University of Salzburg, Salzburg, AUSTRIA; 4 University Clinic of Pneumology, Paracelsus Medical University of Salzburg, Salzburg, AUSTRIA; 5 Sports and Exercise Medicine Division, Department of Medicine, University of Padova, Padova, ITALY; 6 Department of Pulmonary Medicine, Faculty of Medicine, Kepler-University-Hospital, Johannes-Kepler-University, Linz, AUSTRIA; 7 Department of Cardiology, University Heart Center, University Hospital Zurich, Zurich, SWITZERLAND

**Keywords:** CHRONIC OBSTRUCTIVE PULMONARY DISEASE, HYPEROXIA, PULMONARY REHABILITATION, LIMB MUSCLE DYSFUNCTION, QUADRICEPS FEMORIS MUSCLE, MAGNETIC RESONANCE IMAGING

## Abstract

**Objective:**

Exercise training is a cornerstone of the treatment of chronic obstructive pulmonary disease, whereas the related interindividual heterogeneity in skeletal muscle dysfunction and adaptations are not yet fully understood. We set out to investigate the effects of exercise training and supplemental oxygen on functional and structural peripheral muscle adaptation.

**Methods:**

In this prospective, randomized, controlled, double-blind study, 28 patients with nonhypoxemic chronic obstructive pulmonary disease (forced expiratory volume in 1 second, 45.92% ± 9.06%) performed 6 wk of combined endurance and strength training, three times a week while breathing either supplemental oxygen or medical air. The impact on exercise capacity, muscle strength, and quadriceps femoris muscle cross-sectional area (CSA) was assessed by maximal cardiopulmonary exercise testing, 10-repetition maximum strength test of knee extension, and magnetic resonance imaging, respectively.

**Results:**

After exercise training, patients demonstrated a significant increase in functional capacity, aerobic capacity, exercise tolerance, quadriceps muscle strength, and bilateral CSA. Supplemental oxygen affected significantly the training impact on peak work rate when compared with medical air (+0.20 ± 0.03 vs +0.12 ± 0.03 W·kg^−1^, *P* = 0.047); a significant increase in CSA (+3.9 ± 1.3 cm^2^, *P* = 0.013) was only observed in the training group using oxygen. Supplemental oxygen and exercise-induced peripheral desaturation were identified as significant opposing determinants of muscle gain during this exercise training intervention, which led to different adaptations of CSA between the respective subgroups.

**Conclusions:**

The heterogenous functional and structural muscle adaptations seem determined by supplemental oxygen and exercise-induced hypoxia. Indeed, supplemental oxygen may facilitate muscular training adaptations, particularly in limb muscle dysfunction, thereby contributing to the enhanced training responses on maximal aerobic and functional capacity.

Physical exercise training is recommended in all international guidelines on chronic obstructive pulmonary disease (COPD), being an evidence-based central therapeutic measure for these patients (1–5). In this context, improving peripheral muscle structure and function represents a key target for pulmonary rehabilitation, because skeletal muscle mass, strength, and dysfunction were shown to predict mortality independently of lung function (6–10). However, the effects of exercise training on peripheral musculature are still poorly studied and thus not well understood. The magnitude of response to exercise on limb muscles is highly variable, with some patients showing little or no benefit (8,11,12). Indeed, one of the major characteristics of muscle dysfunction in COPD is its interindividual heterogeneity, a fact leading to the concept of a limb muscle dysfunction phenotype in COPD (8). Moreover, characteristic structural and metabolic alterations such as a loss of muscle mass, skeletal muscle fatigue, a shift in fiber type distribution, mitochondrial dysfunction, and decreased oxidative capacity have been described (8,11,13). Despite also chronic cellular hypoxia may affect limb muscle (dys)function (8,14), little is known about the effects of oxygen therapy during exercise on peripheral muscle adaptation in patients with COPD.

It remains unresolved whether nonhypoxemic COPD patients should exercise with supplemental oxygen (5,15–17). Several studies have applied oxygen during endurance exercise training in patients with (18–25) and without exertional hypoxemia (26–29), with conflicting results in both cases. Moreover, it has recently been shown that supplemental oxygen might be able to further improve the effects of endurance training on peak exercise capacity (30). Until this study, little evidence was supporting the use of supplemental oxygen during training interventions in nonhypoxemic COPD (15,16). Technical aspects of oxygen delivery and specific training adaptations must be considered when prescribing supplemental oxygen for pulmonary rehabilitation (28,30–32).

Because supplemental oxygen is known to reduce ventilatory limitation, thereby improving exercise tolerance (33), the hypothesis of this investigation was that supplemental oxygen could help patients to improve and/or accelerate peripheral muscle adaptations/remodeling, leading thereby to better functional capacity. The quadriceps femoris muscle may constitute a valid skeletal muscle to study, because it affects daily physical functioning and represents a typical example of a primary locomotor muscle that is underused in patients with COPD (8). Magnetic resonance imaging (MRI)–based direct lower limb muscle assessment has been shown to provide additional value to objectively evaluate structural peripheral adaptations; indeed, muscle mass correlates with muscle strength and provides useful clinical and functional information (6–8).

In this study, we set out to investigate whether exercise training and supplemental oxygen affect peripheral muscle adaptation, which incorporates important prognostic markers for this population. This is the first training intervention study in patients with COPD, which aims to assess the impact of supplemental oxygen, providing parallel analyses on muscle function and mass evaluated by MRI.

## MATERIALS AND METHODS

**FIGURE 1:**
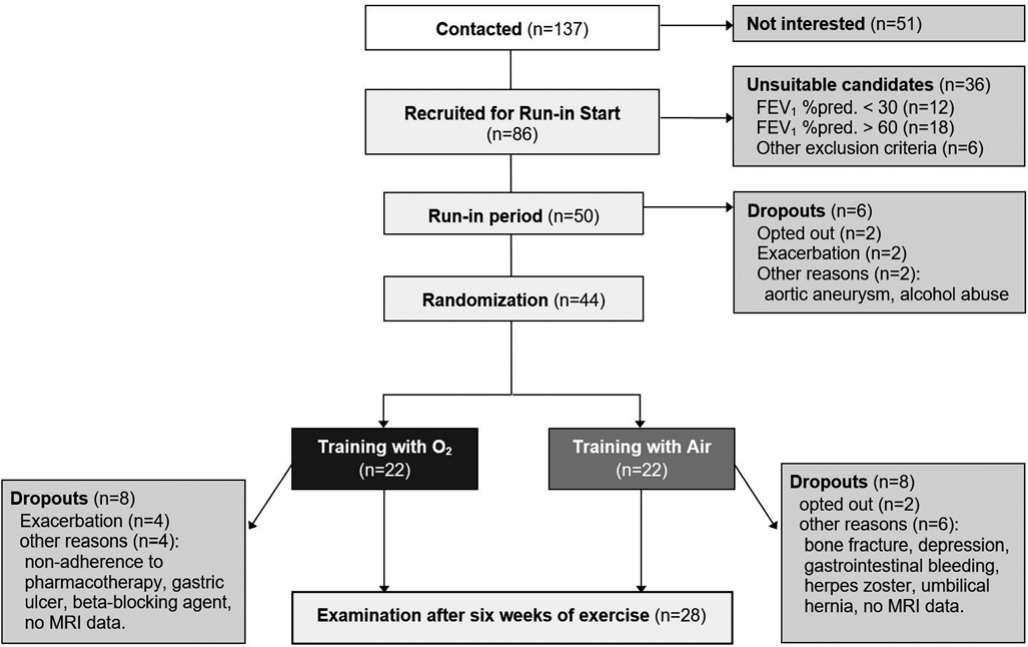
Flow diagram. Fifty of 137 contacted patients met the eligibility criteria and entered the training-free run-in period. At training start, allocation concealment was ensured by an external block randomization for group allocation and concealed medical gas sources by locked away gas cylinders; a hidden gas distributing system was provided from the gas supplier. Although 16 patients could not be evaluated after the training intervention, no differences in dropout rates were observed between both samples. Only two patients opted out during the training period, at a very early stage of the intervention. Other withdrawals were because of comorbidities, exacerbations during the cold winter months, and no available MRI data. The exercise training intervention was conducted at the University Institute of Sports Medicine, Prevention and Rehabilitation of the Paracelsus Medical University of Salzburg (Austria).

The *S*alzburg *COP*D *E*xercise and Oxygen (SCOPE) study is a prospective, randomized, controlled, double-blind, trial in a population with nonhypoxemic COPD (Fig. 1) (30–33). Patients with a stable COPD, aged ≥30 yr, forced expiratory volume in 1 second (FEV_1_) between 30% and 60% of predicted, and resting arterial oxygen partial pressure (PaO_2_) >55 mm Hg, and carbon dioxide partial pressure (PaCO_2_) <45 mm Hg were included; those with comorbidities known to impair physical exercise training were not eligible for participation (30). All included patients underwent a training-free run-in, control period lasting 6 wk to optimize pharmacological treatment according to international guidelines. Subsequently, patients were randomized to training with supplemental oxygen (O_2_) or medical air (Air). Participants, caregivers, and those assessing outcomes were blinded for the provided gas supply. The primary outcome of the SCOPE study assessing the impact of the exercise training intervention and supplemental oxygen was peak work rate (in watts per kilograms) (30). This article specifically provides an analysis of secondary end points of peripheral muscle (dys)function and adaptations to a 6-wk training intervention. Because it was not feasible to perform four MRIs per participant, the crossover analyses of the SCOPE study are not available in this article. The ethics committee of the State of Salzburg approved the study, participants provided written informed consent, and it has been registered on ClinicalTrials.gov (NCT01150383).

### Exercise training intervention

Patients performed 6 wk of combined endurance and strength training three times a week with either supplemental oxygen or medical air during training. Each endurance training session included 5-min warm-up (flow via nasal cannula, 4 L·min^−1^), seven 1-min high-intensity cycling intervals starting at 70%–80% of gas-specific peak work rate each separated by 2 min of active recovery (10 L·min^−1^), and 5-min final cooldown (4 L·min^−1^). The intermittent recovery periods as well as the warm-up and cooldown were performed at about 50% of peak work rate. The exercise intensity in terms of work rate was progressively increased based on training adaptations monitored by heart rate. In addition, eight high-intensity strength training exercises were performed on weight lifting machines with the respective gas supply: latissimus pull-down, shoulder press, back extension, abdominal crunch, butterfly, butterfly reverse, leg extension, and leg flexion. Patients performed 1 set with 8 to 15 repetitions to failure. Whenever more than 15 repetitions were realized, weight was increased.

### Functional evaluation

Functional and aerobic capacity were assessed by ECG-monitored, incremental cardiopulmonary exercise testing (without oxygen supply) using Jaeger Hardware, analyzed with the JLAB Software on a stationary cycle ergometer (Ergoline Ergoselect 200) (30,33). Testing started at 20 W and increased by 10 W·min^−1^ in men and 5 W·min^−1^ in women until exhaustion (Borg rating of perceived exertion: 18–20). Gas exchange parameters were registered breath by breath. Capillary blood samples for lactate measurements and patients’ blood pressure were obtained every 2 min. Lactate measurements were analyzed with the EKF Biosen-C lactate analyzer.

Quadriceps femoris muscle strength was contemporaneously assessed by a standardized 10-repetition maximum (10-RM) strength test of knee extension with Proxomed® compass weight lifting machines. The 10-RM refers to the weight with which the patient was able to perform no more than 10 repetitions.

### Muscle cross-sectional area

**FIGURE 2:**
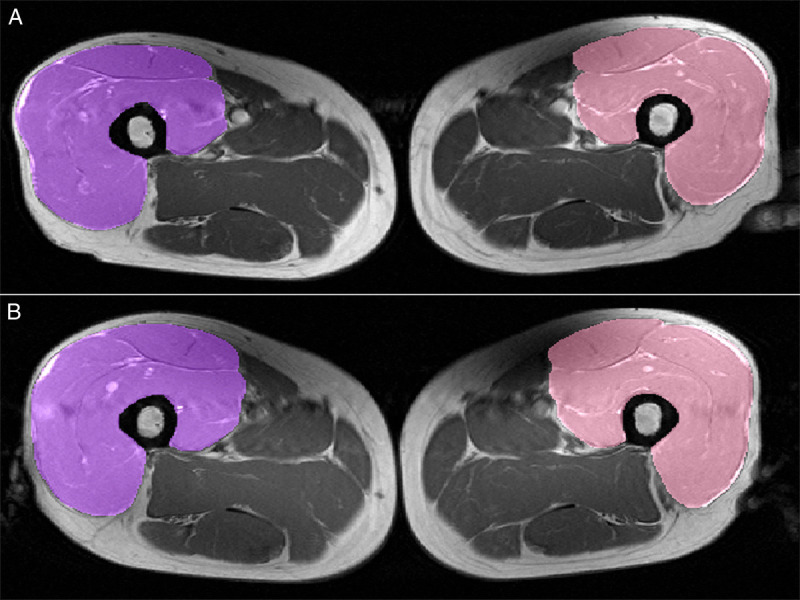
Muscle segmentation in MR images before and after training intervention. T1-weighted MR images with segmentation of the quadriceps femoris muscle (patient’s right side in purple and left side in pink) at baseline (a; top) and after the exercise training intervention (b; bottom). The femur, fatty, and connective tissues as well as vessels were excluded from the segmentation, but fascia lata and beginning rectus tendon were included. This technique and the slice-selection have been shown to adequately represent changes in extensor muscle volume (*r*^2^ = 0.73) (34).

The main outcome of this investigation was the quadriceps femoris muscle cross-sectional area (CSA), assessed by MRI data sets acquired with a 3.0-T scanner (Achieva; Phillips-Medical-Systems, Best, the Netherlands). A T1-weighted turbo-spin-echo sequence (echo time, 15 ms; section thickness, 10 mm; in-plane resolution, 0.78 mm^2^; repetition time, 500–1909 ms; acquisition time, 878–1242 s) was used to obtain axial images. A single representative cross section for both thighs was selected at 30% of distance between the transition of the femoral neck to the shaft and the most distal slice showing the muscular portion of the rectus femoris where it transits to the quadriceps tendon as a singular entity without direct contact to the heads of the muscle. The congruency of the selected cross sections between baseline and follow-up was confirmed by comparing anatomical features like vascular and connective tissue details. The outline of the quadriceps femoris muscle was delineated manually with a custom-made in-house software to obtain the anatomical CSA. The segmentation followed the outline of the quadriceps closely, avoiding surrounding fatty and connective tissues, as well as the fascia lata. However, the quadriceps tendon was included as it is considered part of the muscle (Fig. 2). Reader and quality controller (M.H.) were blinded to acquisition order.

### Statistical analysis

A statistician has been involved in study design and outcome analyses. The Shapiro–Wilk test has been used to test for normality. For normally distributed data, unpaired *t*-tests were performed for comparisons between study arms and paired *t*-tests for analyses within study arms. The Wilcoxon test was used in case of nonnormal data distribution. Subgroup analyses were performed for patients who desaturated during incremental exercise testing (Desat.: peripheral oxygen saturation (SpO_2_) <95%, decrease >5%, measured with pulse oximetry on the fingertip). Comparisons between subgroups were performed with the one-way analysis of variance. A multivariate analysis of variance investigated the impact of supplemental oxygen and exercise-induced desaturation, as determinants of muscle gain during the training intervention. The relationship between continuous variables was evaluated by Pearson’s correlation coefficient. Study outcomes are presented as mean ± SE, whereas baseline characteristics as mean ± SD.

## RESULTS

This study analysis finally evaluated 28 patients aged 63.64 ± 5.97 yr with stable COPD and a mean FEV_1_ of 45.92 ± 9.06% of predicted. The baseline characteristics of the study sample are shown in Table 1, and the study flowchart is presented in Figure 1. At training start, both study groups (supplemental oxygen or medical air) did not differ significantly with regard to pulmonary function (FEV_1_/forced vital capacity, FEV_1_, diffusing capacity of the lung for carbon monoxide, PaCO_2_, PaO_2_, SpO_2_ at rest and peak exercise) and functional capacity, that is, peak work rate (1.1 ± 0.4 vs 1.1 ± 0.4 W·kg^−1^, respectively), peak oxygen uptake (V̇O_2_, 18.2 ± 3.5 vs 18.3 ± 4.0 mL·min^−1^·kg^−1^, respectively), and muscle strength of quadriceps femoris (10-RM, 27.5 ± 9.2 vs 31.3 ± 12.2 kg, respectively; *P* > 0.1 for all parameters).

**TABLE 1:** Baseline characteristics.

Patient Characteristics (*n* = 28; 75% Men)		
	Mean	SD
At rest		
Age, yr	63.6	6.0
BMI, kg·m^−2^	26.9	5.0
FEV_1_/FVC, %	58.6	11.6
FEV_1_, % predicted	45.9	9.1
VC inspired, % predicted	82.0	13.6
DLCOc, % predicted	49.8	12.4
Resting HR, bpm	75.2	15.2
Systolic BP, mm Hg	119.6	14.0
Diastolic BP, mm Hg	73.9	9.2
Hemoglobin, g·dL^−1^	14.9	1.6
PaO_2_, mm Hg	69.3	8.1
PaCO_2_, mm Hg	37.8	3.2
SpO_2_, %	97.0	4.0
At peak exercise		
Peak work rate, W	85.2	31.7
Peak V̇O_2_, mL·kg^−1^·min^−1^	18.2	3.7
VE, L·min^−1^	53.6	11.1
Breathing reserve, %	0.0	16.3
Maximal HR, bpm	129.0	17.0
Systolic BP, mm Hg	177.1	33.6
Diastolic BP, mm Hg	76.8	14.0
BORG, RPE (6–20)	18.8	1.0
Peak RER	1.03	0.07
Maximal lactate, mmol·L^−1^	3.7	1.3
SpO_2_, %	89.9	7.7

This table shows the patients’ baseline characteristics at rest and at peak exercise measured at training start. Data are presented as mean and SD.

BMI, body mass index; BP, blood pressure; Breathing reserve, based on a calculated maximal voluntary ventilation from resting FEV_1_ × 38; DLCOc, diffusing capacity of the lung for carbon monoxide; FVC, forced vital capacity; HR, heart rate; RPE, rating of perceived exertion; VC, vital capacity; VE, minute ventilation.

### Effects of exercise training

Six weeks of exercise training led to a significant increase in functional capacity (peak work rate: 85.3 ± 6.0 vs 97.3 ± 6.2 W, *P* < 0.001; 1.10 ± 0.07 vs 1.25 ± 0.07 W·kg^−1^, *P* < 0.001), aerobic capacity (V̇O_2_, 18.3 ± 0.7 vs 20.1 ± 0.7 mL·min^−1^·kg^−1^, *P* < 0.001), and exercise tolerance (peak respiratory exchange ratio (RER): 1.03 ± 0.01 vs 1.07 ± 0.02, *P* < 0.001; maximal lactate: 3.7 ± 0.2 vs 5.0 ± 0.4 mmol·L^−1^, *P* < 0.001). Furthermore, muscle strength of knee extension increased significantly during the training intervention (10-RM: 29.5 ± 2.1 vs 41.3 ± 2.2 kg, *P* < 0.001). A growth in CSA has been observed for both quadriceps femoris muscles (left CSA: 57.6 ± 2.5 vs 58.6 ± 2.5 cm^2^, *P* = 0.049; right CSA: 56.7 ± 2.6 vs 57.7 ± 2.7 cm^2^, *P* = 0.042; Fig. 2).

### Effects of supplemental oxygen

Although both study groups significantly improved their maximal exercise capacity, when both breathing conditions were compared after 6 wk of exercise intervention, it was observed that supplemental oxygen positively affected peak work rate when compared with medical air (differences: O_2_ +15.1 ± 2.3 vs Air +9.5 ± 1.9 W, *P* = 0.067; O_2_ +0.20 ± 0.03 vs air +0.12 ± 0.03 W·kg^−1^, *P* = 0.047). This impact on functional capacity was also associated with structural adaptations in peripheral muscles. Although those patients who trained with supplemental oxygen were able to significantly increase the total quadriceps femoris CSA (+3.9 ± 1.3 cm^2^, *P* = 0.013), this was not the case during training with medical air (+0.6 ± 1.3 cm^2^, *P* = 0.656). However, the increase in muscle strength of knee extension was not found significantly affected by supplemental oxygen (differences: O_2_ +11.1 ± 1.1 vs Air +12.3 ± 2.2 kg, *P* = 0.649).

### Impact of exercise-induced peripheral desaturation

Although those patients who did not desaturate during exercise were able to significantly increase their total quadriceps femoris CSA during the training intervention (+4.0 ± 1.4 cm^2^, *P* = 0.012), no structural peripheral adaptation was found for the subgroup with exercise-induced peripheral desaturation (54% of the study participants; +0.5 ± 1.3 cm^2^, *P* = 0.701).

### Subgroup analyses

**FIGURE 3:**
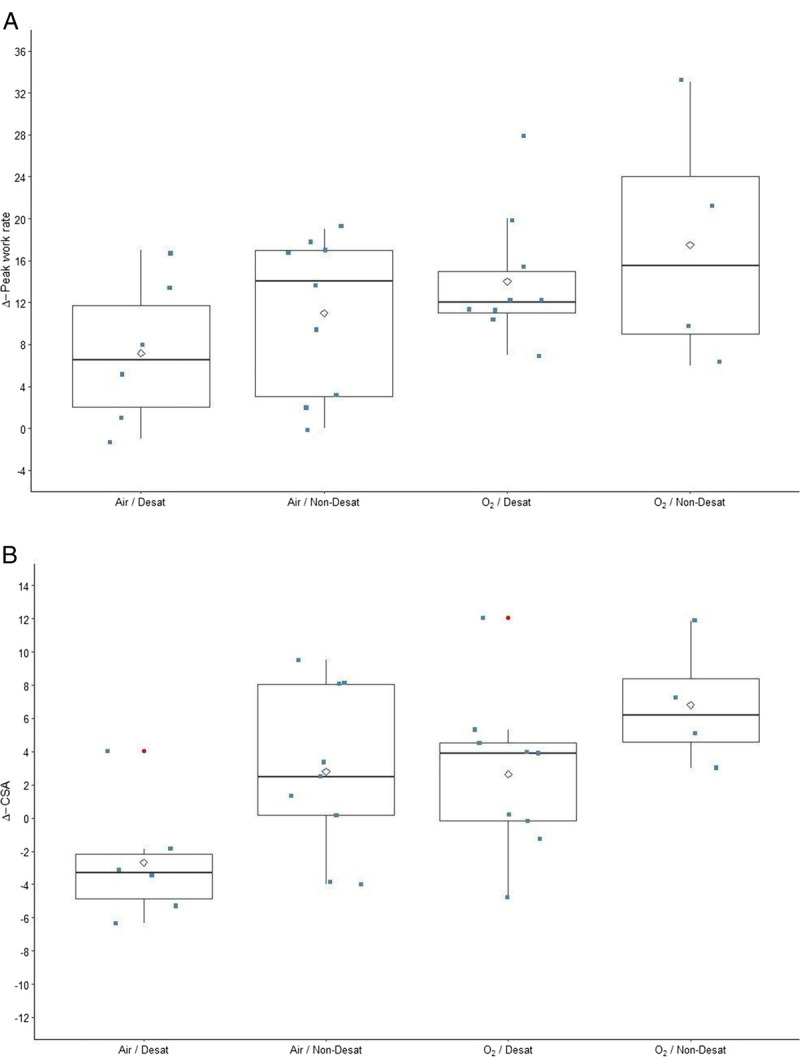
Subgroup comparisons of exercise capacity and CSA. The changes (Δ) of peak work rate (in watts) and quadriceps femoris muscle CSA (in centimeters squared) are presented in panels a and b, respectively. Functional and structural adaptation showed both similar training responses in the different subgroups.

**FIGURE 4:**
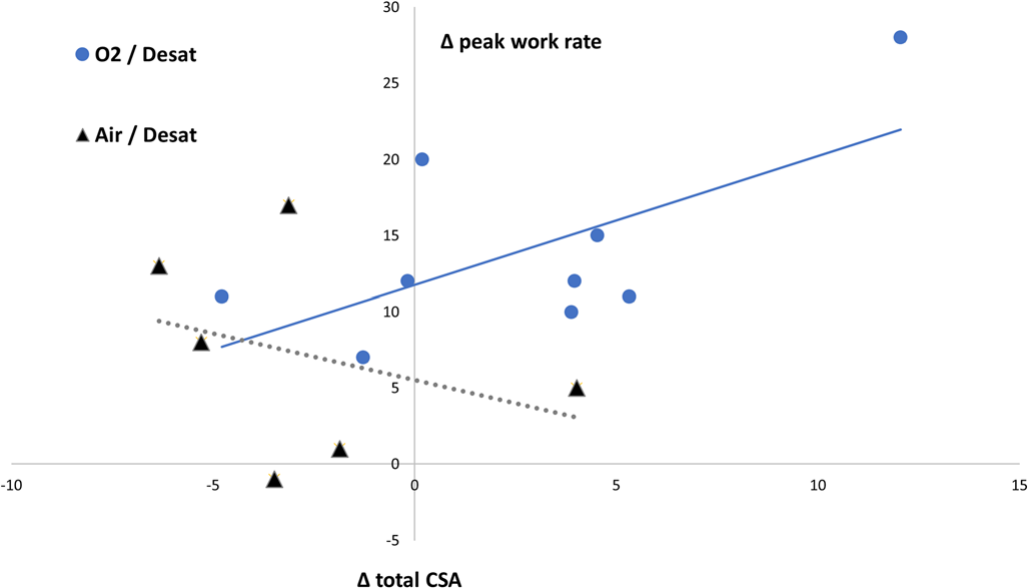
Impact of supplemental oxygen on training adaptations in desaturating patients. The impact of supplemental oxygen on patients with exercise-induced peripheral desaturation significantly affected the training adaptations on quadriceps femoris CSA (ΔCSA [in centimeters squared]) related to the respective peak work rate (Δ [in watts]).

The impact of supplemental oxygen and exercise-induced peripheral desaturation led to different adaptations of total quadriceps femoris CSA between the respective subgroups (Table 2); a multivariate analysis of variance showed that both were significant opposing determinants of muscle gain during the exercise training intervention (*P* = 0.005 and *P* = 0.008, respectively). Moreover, peak work rate and quadriceps femoris muscle CSA showed independently similar training adaptations in the different subgroups (Fig. 3). The nonbenefit of the exercise training intervention on CSA in desaturating patients training with medical air should be highlighted, whereas supplemental oxygen was able to compensate this missed peripheral adaptation (Fig. 4). The opposite impact of supplemental oxygen and exercise-induced peripheral desaturation affected training adaptations in peripheral muscles and thus influenced peak work rate. This was confirmed by significant correlations of the changes in total CSA with the impact on functional capacity and exercise tolerance during training with supplemental oxygen; these correlations were found inverted for patients with exercise-induced peripheral desaturation who trained with medical air (Table 3, Fig. 4).

**TABLE 2:** Impact of exercise training on quadriceps femoris muscle CSA.

	Subgroups	Training Start			Training End			Difference		*P*
		*n*	Mean	SE	*n*	Mean	SE	Mean	SE	
Total CSA	All	28	114.2	5.1	28	116.4	5.1	2.1	1.0	0.038
	Air/Desat	6	119.1	9.5	6	116.4	9.5	−2.7	1.4	0.049
	Air/Non-Desat	9	121.1	9.4	9	123.9	9.0	2.8	1.7	
	O_2_/Desat	9	111.0	8.3	9	113.6	8.8	2.6	1.6	
	O_2_/Non-Desat	4	98.6	18.4	4	105.4	20	6.8*	1.9	

This table shows that, although patients’ total CSA (in centimeters squared) of their quadriceps femoris muscle has increased during the exercise training intervention, the impact was found statistically significant different when subgroups were compared with a one-way analysis of variance (*P* = 0.049).

*P < 0.05 for intragroup comparison.

**TABLE 3:** Correlations of structural with functional parameters of exercise capacity.

	Subgroups	*n*	Peak Work Rate, W	Peak Work Rate, W·kg^−1^	Peak V̇O_2_, mL·kg^−1^·min^−1^	10-RM Knee Extension, kg	Peak RER	Peak Lactate, mmol·L^−1^
Differences total CSA	All	28	0.489**	0.399*	0.267	0.256	0.414*	0.323
	O_2_	13	0.594*	0.432	0.668 *	0.228	0.651*	0.672*
	Air	15	0.277	0.231	−0.107	0.351	0.204	−0.158
	Desat	15	0.487	0.213	0.339	0.247	0.264	0.425
	Non-Desat	13	0.491	0.503	0.304	0.305	0.588*	0.287
	Air/Desat	6	−0.323	−0.266	−0.361	0.306	−0.467	−0.601
	Air/Non-Desat	9	0.371	0.352	0.164	0.474	0.469	0.014
	O_2_/Desat	9	0.644*	0.188	0.731*	0.475	0.570	0.717*
	O_2_/Non-Desat	4	0.588	0.504	0.291	−0.482	0.962*	0.818

This table provides an overview on how the changes of total CSA (quadriceps femoris muscle) during the training intervention are correlated with the impact on functional capacity, aerobic capacity, muscle strength, and exercise tolerance. Pearson’s correlation coefficient is provided for all variables.

*P < 0.05.

**P < 0.01.

## DISCUSSION

To the best of our knowledge, this is the first study to investigate the impact of an exercise training intervention with and without supplemental oxygen on independently assessed end points of functional and structural peripheral muscle adaptations in patients with nonhypoxemic COPD. The innovative assessment of the quadriceps femoris muscle CSA by MRI provides new insights into the training adaptations during pulmonary rehabilitation. In this population, limb muscle dysfunction and the related interindividual variability in the effects of exercise training on skeletal muscles are not yet fully understood. The main results of this investigation suggest the following interpretations:

- Peripheral functional and structural training adaptations are highly variable in this population.
- Supplemental oxygen increases the impact of exercise training on peak work rate and skeletal muscle mass.
- Peripheral muscle fatigue and hypoxia during exercise may affect the training response on muscle gain.
- Exercise-induced intermittent hypoxia might be a determinant to further investigate regarding its association with limb muscle dysfunction in COPD.

### Impact of exercise training and supplemental oxygen

Exercise training led to a significant increase in functional capacity, aerobic capacity, and exercise tolerance. Furthermore, a gain in muscle strength of knee extension has been shown after the training intervention. Data also suggest an important role of supplemental oxygen during exercise training, which significantly increased the functional training effects on peak exercise intensities (30). Although oxygen supplementation during training may have limited direct effects on muscle oxygenation in certain conditions/patients, it also inhibits carotid body stimulation and reduces thereby respiratory drive (30,35,36). Moreover, it is known that supplemental oxygen can improve pulmonary hemodynamics by local vasodilatation, leading to lower pulmonary resistances and thus increased cardiac output, allowing thereby better exercise tolerance during training sessions (37). Thus, supplemental oxygen could reduce ventilatory limitation during exercise and permit higher training loads/volume, also because of greater peripheral muscle activation and less fatigability (13), consequently leading to improved exercise capacity due to better training stimuli (28,30,33). Until publication of the results of our SCOPE study supporting the use of supplemental oxygen during training in nonhypoxemic COPD, the outcomes from previous studies were rather heterogenous (15–17). Different study designs and technical aspects of oxygen delivery might have contributed to the inconclusive discussion (16). High oxygen flows, gas-specific exercise prescription, higher exercise intensities, and progressive training adaptations were proposed as critical issues (15,30,33,36). However, the impact of supplemental oxygen seems more evident on maximal workloads than on submaximal exercise capacity (25,30,32). Furthermore, the effects of exercise training on endothelial dysfunction and chronic inflammation were not significantly affected by supplemental oxygen (31). All these aspects might raise the question if the study hypothesis regarding supplemental oxygen during training should be reconsidered; from “whether or not” toward “when, how, for which training objectives and patients.”

### Peripheral muscular training adaptation

This study provides additional value in evaluating the impact of supplemental oxygen during training in patients with COPD, because the previously discussed functional improvements on exercise capacity were associated with independently obtained parameters of peripheral muscular training adaptation (30). Skeletal muscle dysfunction and strength are predictors of mortality independent of lung function (7–9), and the related most potent currently available treatment option is exercise training (8,10,11). Strength tests and evaluation of skeletal muscle mass could thus provide useful clinical and prognostic information for patients with COPD. MRI-based direct lower limb muscle assessment objectively evaluates structural peripheral adaptation, which is particularly useful in research because these methods may be more responsive to specific lower limb interventions (8,38). Indeed, the study sample’s CSAs of the quadriceps femoris muscles increased significantly in 6 wk of training. However, subgroup analyses showed that, only during training with supplemental oxygen, a significant gain in muscle mass could be obtained. The impact of supplemental oxygen on patients’ muscle growth was particularly apparent for the subgroup with exercise-induced peripheral desaturation. Moreover, those patients of this subgroup who have trained with medical air did not show any improvement with a trend of decreasing the CSA of the quadriceps femoris muscles. On the other hand, supplemental oxygen led to a clear mitigation of this maladaptation and thus to an increase in muscle CSA and improved peak work rate (Fig. 4). Indeed, therapies, including those aimed at alleviating hypoxia, have been shown to partially restore muscle mass and oxidative capacity in patients with chronic respiratory disease (39). Although many COPD patients may not experience excessive tissue hypoxia during standard pulmonary rehabilitation programs, it was shown that hypoxia can be a key factor driving changes in limb muscle tissue, affecting muscle weakness, dysfunction, and atrophy through altered signalization in the HIF/von Hippel–Lindau pathway as well as by epigenetic mechanisms (8,14,40). Diseased muscles are largely unable to coordinate the expression of muscle remodeling (40); hypoxia has an inhibitory effect on muscle protein synthesis and leads to an activation of proteolysis (8,14). Hypoxemia may also potentiate the inflammatory response and compromise muscles’ oxidative capacity, providing additional mechanisms predisposing to muscle atrophy and fatigue (8,39). The SCOPE trial cannot provide direct mechanistic data confirming these pathophysiological (mal)adaptations in skeletal muscles, but study outcomes might emphasize the impact supplemental oxygen could have on limb muscle adaptations to prevent the consequences of exercise-induced hypoxia in certain patients with COPD. The fact that this hypothesis has not been confirmed, evaluating muscle strength of knee extension could be due to the important exercise-specific neuromuscular learning effect during strength training, which may have a relatively greater impact than changes in CSA in a short training period for patients who are not used to strength exercises. Future trials may reassess the effect of hypoxia/hyperoxia on strength gain during longer training interventions, with different training modalities and a higher volume of strength exercises, providing also follow-up evaluations and accurate isokinetic/isometric strength analyses.

### Limb muscle dysfunction in COPD

The interindividual heterogeneity regarding muscle dysfunction, weakness, and adaptation in these patients led to the concept that an unexpected limb muscle dysfunction phenotype may exist in COPD (8). Furthermore, although pulmonary rehabilitation improves limb muscle function (4), it has been shown that the magnitude of response to exercise training interventions is highly variable, with some patients showing little or no benefit (12). Clusters of COPD patients with differential muscle molecular rehabilitation responses have been described, which may be reflective of distinct phases of muscle remodeling and correspond to a differential gain in physical functioning (11). The data of our study are in line with previous publications, which discussed a high interindividual variability of training-related functional and structural peripheral muscle adaptations (8,11,12). This is mirrored by significant differences between subgroups, revealing supplemental oxygen and exercise-induced peripheral desaturation as significant opposing determinants of skeletal muscle adaptation. Although the development of limb muscle dysfunction is related to multifactorial issues like physical inactivity, chronic inflammation, nutritional imbalance, medical drugs, hypercapnia, and oxidative stress, also hypoxemia has been discussed as possible trigger contributing to peripheral structural degradation (8,14). Thus, our study observations may suggest that (exercise-induced intermittent) hypoxia could significantly influence limb muscle adaptations and dysfunction, which would also partially explain the substantial interindividual variability in the effects of pulmonary rehabilitation on skeletal muscles (11). These outcomes add further pathophysiological and mechanistic hypotheses how supplemental oxygen may affect a training intervention, providing also data related to its impact on peripheral muscle mass, which was independently evaluated from functional testing. This should motivate further studies to specifically investigate the impact of supplemental oxygen on skeletal muscles regarding structural, cellular, and molecular adaptations in different COPD subpopulations and phenotypes.

### Limitations and perspectives

A limitation of this study is that MRI repetition and acquisition times varied because of software updates of the scanner in between. However, it has been shown that different turbo-spin-echo sequences, acquired at different scanners, and even with different echo times will yield highly reproducible shape parameters (41). The muscle CSA analysis is exclusively morphological, and no quality measures like spectroscopy were performed. However, it has been shown that muscle CSA correlates strongly with muscle quality measures (42). Furthermore, because of the complexity of conducting RCTs in such COPD patients, the statistical power of this secondary analysis of the SCOPE study limits strong conclusions for subgroup analyses with small sample size. However, because functional and structural peripheral muscle adaptations showed very similar results by independent markers and evaluations, the risk of bias in the study interpretation seems rather low. Further specific research with larger prospective cohorts and a well-characterized COPD population is needed to confirm these outcomes and to improve existing knowledge regarding the pathophysiological mechanisms of limb muscle dysfunction and the related muscular adaptations to exercise. Because this study cannot provide mechanistic data supporting the hypothesis of cellular hypoxia as determinant of skeletal muscle adaptations during an exercise training intervention, direct measurements of peripheral oxygen supply to muscles with an assessment of molecular and cellular training adaptations may carry this research question forward. Future studies should add analyses of muscle proteomics/metabolomics with muscle biopsies and/or specific imaging techniques; also measures of electromyography should be investigated to identify whether the enhanced training adaptation is consequent to increased muscle activity. This may improve the understanding of the heterogenous peripheral training adaptations in skeletal muscles of patients with COPD.

## CONCLUSIONS

To the best of our knowledge, this is the first randomized, controlled, double-blind trial to provide an evaluation of functional and morphological adaptations of thigh muscles after an exercise intervention with and without supplemental oxygen during training in patients with COPD. Supplemental oxygen significantly increased the training impact on peak work rate and fostered quadriceps femoris muscle growth. Our data may generate the hypothesis that exercise-induced peripheral hypoxia limits muscular training adaptations, which might, at least partially, explain the high interindividual variability, supporting the concept of a possible limb muscle dysfunction phenotype in COPD. However, this could be mitigated by providing supplemental oxygen during exercise training, which demonstrated great importance to ensure muscle gain and thus improve peak exercise capacity. Although the current evidence for providing supplemental oxygen during training for patients with nonhypoxemic COPD is limited, future studies should not anymore focus on the yes or no, but they may aim to understand when, how, and in which patients it could be best used.

## References

[1] Mccarthy B Casey D Devane D Murphy K Murphy E Lacasse Y. Pulmonary rehabilitation for chronic obstructive pulmonary disease. *Cochrane Database Syst Rev*. 2015;2015(2):CD003793. 25705944 10.1002/14651858.CD003793.pub3 PMC10008021

[2] Watz H Pitta F Rochester CL, . An official European Respiratory Society statement on physical activity in COPD. *Eur Respir J*. 2014;44(6):1521–37. 25359358 10.1183/09031936.00046814

[3] Singh D Agusti A Anzueto A, . Global strategy for the diagnosis, management, and prevention of chronic obstructive lung disease: the GOLD science committee report 2019. *Eur Respir J*. 2019;53(5):1900164. 30846476 10.1183/13993003.00164-2019

[4] Spruit MA Pitta F McAuley E ZuWallack RL Nici L. Pulmonary rehabilitation and physical activity in patients with chronic obstructive pulmonary disease. *Am J Respir Crit Care Med*. 2015;192(8):924–33. 26161676 10.1164/rccm.201505-0929CI

[5] Spruit MA Singh SJ Garvey C, . An official American Thoracic Society/European Respiratory Society statement: key concepts and advances in pulmonary rehabilitation. *Am J Respir Crit Care Med*. 2013;188(8):e13–64. 24127811 10.1164/rccm.201309-1634ST

[6] Marillier M Bernard AC Vergès S Neder JA. Locomotor muscles in COPD: the rationale for rehabilitative exercise training. *Front Physiol*. 2020;10:1590. 31992992 10.3389/fphys.2019.01590 PMC6971045

[7] Marquis K Debigaré R Lacasse Y, . Midthigh muscle cross-sectional area is a better predictor of mortality than body mass index in patients with chronic obstructive pulmonary disease. *Am J Respir Crit Care Med*. 2002;166(6):809–13. 12231489 10.1164/rccm.2107031

[8] Maltais F Decramer M Casaburi R, . An official American thoracic society/european respiratory society statement: update on limb muscle dysfunction in chronic obstructive pulmonary disease. *Am J Respir Crit Care Med*. 2014;189(9):e15–62. 24787074 10.1164/rccm.201402-0373ST PMC4098112

[9] Swallow EB Reyes D Hopkinson NS, . Quadriceps strength predicts mortality in patients with moderate to severe chronic obstructive pulmonary disease. *Thorax*. 2007;62(2):115–20. 17090575 10.1136/thx.2006.062026 PMC2111256

[10] Li P Li J Wang Y Xia J Liu X. Effects of exercise intervention on peripheral skeletal muscle in stable patients with COPD: a systematic review and meta-analysis. *Front Med (Lausanne)*. 2021;18(8):766841. 10.3389/fmed.2021.766841 PMC8636927 34869477

[11] Kneppers AEM Haast RAM Langen RCJ, . Distinct skeletal muscle molecular responses to pulmonary rehabilitation in chronic obstructive pulmonary disease: a cluster analysis. *J Cachexia Sarcopenia Muscle*. 2019;10(2):311–22. 30657653 10.1002/jcsm.12370 PMC6463471

[12] Troosters T Gosselink R Decramer M. Exercise training in COPD: how to distinguish responders from nonresponders. *J Cardiopulm Rehabil*. 2001;21(1):10–7. 11271652 10.1097/00008483-200101000-00004

[13] Cannon DT Coelho AC Cao R, . Skeletal muscle power and fatigue at the tolerable limit of ramp-incremental exercise in COPD. *J Appl Physiol (1985)*. 2016;121(6):1365–73. 27660300 10.1152/japplphysiol.00660.2016

[14] Abdulai RM Jensen TJ Patel NR, . Deterioration of limb muscle function during acute exacerbation of chronic obstructive pulmonary disease. *Am J Respir Crit Care Med*. 2018;197(4):433–49. 29064260 10.1164/rccm.201703-0615CI PMC5821903

[15] Nonoyama M Brooks D Lacasse Y Gh G Goldstein R. Oxygen therapy during exercise training in chronic obstructive pulmonary disease. *Cochrane Database Syst Rev*. 2007;18(2):CD005372. 10.1002/14651858.CD005372.pub2 PMC8885311 17443585

[16] Freitag N Doma K Neunhaeuserer D Cheng S Bloch W Schumann M. Is structured exercise performed with supplemental oxygen a promising method of personalized medicine in the therapy of chronic diseases? *J Pers Med*. 2020;10(3):135. 32961816 10.3390/jpm10030135 PMC7564446

[17] Liu Y Gong F. Determination of whether supplemental oxygen therapy is beneficial during exercise training in patients with COPD: a systematic review and meta-analysis. *Exp Ther Med*. 2019;18(5):4081–9. 31616520 10.3892/etm.2019.8026 PMC6781835

[18] Rooyackers JM Dekhuijzen PN van Herwaarden CL Folgering HT. Training with supplemental oxygen in patients with COPD and hypoxaemia at peak exercise. *Eur Respir J*. 1997;10(6):1278–84. 9192929 10.1183/09031936.97.10061278

[19] Garrod R Paul EA Wedzicha JA. Supplemental oxygen during pulmonary rehabilitation in patients with COPD with exercise hypoxaemia. *Thorax*. 2000;55(7):539–43. 10856310 10.1136/thorax.55.7.539 PMC1745814

[20] Mcdonald CF Blyth CM Lazarus MD Marschner IAN Barter CE. Exertional oxygen of limited benefit in patients with chronic obstrudive pulmonary disease and mild hypoxemia. *Am J Respir Crit Care Med*. 1995;152(5 Pt 1):1616–9. 7582304 10.1164/ajrccm.152.5.7582304

[21] Ringbaek T Martinez G Lange P. The long-term effect of ambulatory oxygen in normoxaemic COPD patients: a randomised study. *Chron Respir Dis*. 2013;10(2):77–84. 23431028 10.1177/1479972312473135

[22] Wadell K Henriksson-Larsen K Lundgren R. Physical training with and without oxygen in patients with chronic obstructive pulmonary disease and exercise-induced hypoxaemia. *J Rehab Med*. 2001;33(24):200–5. 10.1080/165019701750419581 11585150

[23] Helgerud J Bjørgen S Karlsen T, . Hyperoxic interval training in chronic obstructive pulmonary disease patients with oxygen desaturation at peak exercise. *Scand J Med Sci Sports*. 2010;20(1):e170–6. 19793218 10.1111/j.1600-0838.2009.00937.x

[24] Eaton T Garrett JE Young P, . Ambulatory oxygen improves quality of life of COPD patients: a randomised controlled study. *Eur Respir J*. 2002;20(2):306–12. 12212960 10.1183/09031936.02.00301002

[25] Alison JA McKeough ZJ Leung RWM, . Oxygen compared to air during exercise training in COPD with exercise-induced desaturation. *Eur Respir J*. 2019;53(5):1802429. 30880289 10.1183/13993003.02429-2018

[26] Scorsone D Bartolini S Saporiti R, . Does a low-density gas mixture or oxygen supplementation improve exercise training in COPD? *Chest*. 2010;138(5):1133–9. 20495110 10.1378/chest.10-0120

[27] Spielmanns M Fuchs-Bergsma C Winkler A Fox G Krüger S Baum K. Effects of oxygen supply during training on subjects with COPD who are normoxemic at rest and during exercise: a blinded randomized controlled trial. *Respir Care*. 2015;60(4):540–8. 25516993 10.4187/respcare.03647

[28] Emtner M Porszasz J Burns M Somfay A Casaburi R. Benefits of supplemental oxygen in exercise training in nonhypoxemic chronic obstructive pulmonary disease patients. *Am J Respir Crit Care Med*. 2003;168(9):1034–42. 12869359 10.1164/rccm.200212-1525OC

[29] Eves ND Sandmeyer LC Wong EY, . Helium-hyperoxia: a novel intervention to improve the benefits of pulmonary rehabilitation for patients with COPD. *Chest*. 2009;135(3):609–18. 19017883 10.1378/chest.08-1517

[30] Neunhäuserer D Steidle-Kloc E Weiss G, . Supplemental oxygen during high intensity exercise training in nonhypoxemic COPD. *Am J Med*. 2016;129(11):1185–93. 27427325 10.1016/j.amjmed.2016.06.023

[31] Neunhäuserer D Patti A Niederseer D, . Systemic inflammation, vascular function, and endothelial progenitor cells after an exercise training intervention in COPD. *Am J Med*. 2021;134(3):e171–80. 32781050 10.1016/j.amjmed.2020.07.004

[32] Neunhäuserer D Reich B Mayr B, . Impact of exercise training and supplemental oxygen on submaximal exercise performance in patients with COPD. *Scand J Med Sci Sports*. 2021;31(3):710–9. 33155295 10.1111/sms.13870 PMC7984048

[33] Neunhäuserer D Steidle-Kloc E Bergamin M, . Role of breathing conditions during exercise testing on training prescription in chronic obstructive pulmonary disease. *Am J Phys Med Rehabil*. 2017;96(12):908–11. 28644243 10.1097/PHM.0000000000000775

[34] Cotofana S Hudelmaier M Wirth W, . Correlation between single-slice muscle anatomical cross-sectional area and muscle volume in thigh extensors, flexors and adductors of perimenopausal women. *Eur J Appl Physiol*. 2010;110(1):91–7. 20401666 10.1007/s00421-010-1477-8

[35] Somfay A Porszasz J Lee S Casaburi R. Effect of hyperoxia on gas exchange and lactate kinetics following exercise onset in nonhypoxemic COPD patients. *Chest*. 2002;121(2):393–400. 11834648 10.1378/chest.121.2.393

[36] Bitos K Furian M Mayer L, . Effect of high-flow oxygen on exercise performance in COPD patients. randomized trial. *Front Med (Lausanne)*. 2021;19(7):595450. 10.3389/fmed.2020.595450 PMC7938234 33693009

[37] Fujimoto K Matsuzawa Y Yamaguchi S Koizumi T Kubo K. Benefits of oxygen on exercise performance and pulmonary hemodynamics in patients with COPD with mild hypoxemia. *Chest*. 2002;122(2):457–63. 12171817 10.1378/chest.122.2.457

[38] HajGhanbari B Hamarneh G Changizi N Ward AD Reid WD. MRI-based 3D shape analysis of thigh muscles: patients with chronic obstructive pulmonary disease versus healthy adults. *Acad Radiol*. 2011;18(2):155–66. 21111639 10.1016/j.acra.2010.09.008

[39] Theije C de Costes F Langen RC Pison C Gosker HR. Hypoxia and muscle maintenance regulation: implications for chronic respiratory disease. *Curr Opin Clin Nutr Metab Care*. 2011;14(6):548–53. 21934612 10.1097/MCO.0b013e32834b6e79

[40] Turan N Kalko S Stincone A, . A systems biology approach identifies molecular networks defining skeletal muscle abnormalities in chronic obstructive pulmonary disease. *PLoS Comput Biol*. 2011;7(9):e1002129. 21909251 10.1371/journal.pcbi.1002129 PMC3164707

[41] Wong OL Yuan J Zhou Y Yu SK Cheung KY. Longitudinal acquisition repeatability of MRI radiomics features: an ACR MRI phantom study on two MRI scanners using a 3D T1W TSE sequence. *Med Phys*. 2021;48(3):1239–49. 33370474 10.1002/mp.14686

[42] Otsuka Y Yamada Y Maeda A, . Effects of resistance training intensity on muscle quantity/quality in middle-aged and older people: a randomized controlled trial. *J Cachexia Sarcopenia Muscle*. 2022;13(2):894–908. 35187867 10.1002/jcsm.12941 PMC8977953

